# Embodied Computational Evolution: Feedback Between Development and Evolution in Simulated Biorobots

**DOI:** 10.3389/frobt.2021.674823

**Published:** 2021-06-10

**Authors:** Joshua Hawthorne-Madell, Eric Aaron, Ken Livingston, John H. Long

**Affiliations:** ^1^ Interdisciplinary Robotics Research Laboratory, Vassar College, Poughkeepsie, NY, United States; ^2^ Department of Cognitive Science, Vassar College, Poughkeepsie, NY, United States; ^3^ Department of Computer Science, Colby College, Waterville, ME, United States

**Keywords:** embodied computational evolution, development, epigenetics, genetic variation, population genetics

## Abstract

Given that selection removes genetic variance from evolving populations, thereby reducing exploration opportunities, it is important to find mechanisms that create genetic variation without the disruption of adapted genes and genomes caused by random mutation. Just such an alternative is offered by random epigenetic error, a developmental process that acts on materials and parts expressed by the genome. In this system of embodied computational evolution, simulated within a physics engine, epigenetic error was instantiated in an explicit genotype-to-phenotype map as transcription error at the initiation of gene expression. The hypothesis was that transcription error would create genetic variance by shielding genes from the direct impact of selection, creating, in the process, *masquerading genomes*. To test this hypothesis, populations of simulated embodied biorobots and their developmental systems were evolved under steady directional selection as equivalent rates of random mutation and random transcriptional error were covaried systematically in an 11 × 11 fully factorial experimental design. In each of the 121 different experimental conditions (unique combinations of mutation and transcription error), the same set of 10 randomly created replicate populations of 60 individuals were evolved. Selection for the improved locomotor behavior of individuals led to increased mean fitness of populations over 100 generations at nearly all levels and combinations of mutation and transcription error. When the effects of both types of error were partitioned statistically, increasing transcription error was shown to increase the final genetic variance of populations, incurring a fitness cost but acting on variance independently and differently from genetic mutation. Thus, random epigenetic errors in development feed back through selection of individuals with masquerading genomes to the population’s genetic variance over generational time. Random developmental processes offer an additional mechanism for exploration by increasing genetic variation in the face of steady, directional selection.

## Introduction

Understanding the evolutionary dynamics of embodied^1^, locomoting individuals—whether organic, living creatures or mechanical, manufactured robots—is of central importance to studies of living systems and robotic systems alike. The full span of factors and subsystems underlying biological or bio-inspired evolution, however, cannot feasibly be fully studied solely by experiments with living organisms (Long, 2012). Consider, for example, the processes underlying the genotype-to-phenotype map (G-P map, for short), the developmental processes by which an individual's physical form is constructed from its genetic encoding (Bongard, 2002; Wagner, 2005). Mechanisms within the processes of transcribing the genotype—not themselves elements of the genome nor the embodied phenotype, but extra-genetic or epigenetic processes^2^ that act on genes in the G-P map—could have effects on the locomotion of individuals or on the genetic composition of a population. Controlled computational experimentation could yield novel insights into living systems, novel mechanisms for inclusion in the methodologies of evolutionary robotics (Doncieux et al., 2015), or both.

In this paper, we present our methodology for investigating the role of randomness in the G-P map of evolving, locomoting individuals, and we present the first results of our experiments with developing, evolving, simulated robots. In particular, our results include support for a *masquerading genome hypothesis* (MGH): The presence of noise in the gene transcription process of the G-P map can shield genes from the direct impact of selection, thus increasing genetic variance in populations over generational time. Moreover, the MGH is distinct from genetic mutation, acting independently and differently on variance. There is, however, a fitness cost that accompanies the increased variance resulting from masquerading genomes. These results typify *embodied computational evolution (ECE)*, the employment and study of evolutionary methods using embodied robots, virtual or material—an approach distinct from other evolutionary computation approaches, for which embodiment is not integral. These ECE results provide novel insight into living systems using computational methods; these results may also be of interest to evolutionary robotics, for which increased variance may help avoid convergence on local optima (Pugh et al., 2015; Pugh et al., 2016), although as we discuss in the Discussion, the fitness cost may not always be worth incurring for the added variance.

In organisms, G-P mappings begin with the expression of the genetic code: triplet codons are modeled canonically as redundant with respect to the intended amino acid and hence are neutral at the position of the third base pair with regard to phenotype (for an alternate model, see (Spencer and Barral, 2012)). The redundancy of the genetic code is one way to create neutral genetic variation. Neutral effects with respect to phenotypic expression may also occur for most other genetic levels and effects (Paaby and Rockman, 2014), a general phenomenon called cryptic genetic variation (CGV), which may serve as a genetic reservoir for future evolutionary change (Zheng et al., 2019). In populations of bacteria, CGV facilitated adaptation when the population faced rapid environmental change as enacted by a change in selection (Lee and Marx, 2019; Zheng et al., 2019).

As the “cryptic” in its name denotes, CGV increases genetic variation by “hiding” genes from selection; in the simplest case, if genes aren’t expressed—a process that begins with the transcription of DNA into messenger RNA—then they cannot be selected. Thus CGV aids diversification in the long run by decoupling a subset of the connections in the G-P map. For the connections that remain, their mappings can be altered by epigenetic processes. When noise is introduced into the G-P map for the neurocontrollers of simulated quadrupedal robots, the resulting stochastic ontogenesis (SO), a developmental process, can have surprising and positive evolutionary consequences (Lee and Marx, 2019). As is true with populations possessing CGV, populations with SO respond better, as measured by evolutionary fitness, to changes in the environment, apparently by providing a reservoir of solutions. In order to transfer the reservoir of solutions from SO over generational time, those solutions must have a genetic basis, as indicated by the presence of higher scoring replicates in SO populations (Stanton, 2018).

Thus we hypothesize that the epigenetic SO mechanism increases genetic variation in populations under selection, hiding some genes from selection in a manner that is functionally similar to CGV. To test this hypothesis, we evolve populations of simulated embodied robots under directional selection, covarying levels of genetic noise created by random mutation with levels of epigenetic noise created by random transcriptional error. We then select for improved locomotor behavior while tracking both genetic and phenotypic variation in the populations.

We chose to manipulate the bioinspired mechanism of transcription because it is the first and arguably the most important stage of gene expression and development. In the context of evolutionary robotics, transcription has been used as an explicit factor in body-brain evolution (Krčah, 2012) and transcription errors have been hypothesized to overcome premature convergence caused by selection (Borg et al., 2011; Borg, 2018). In biological organisms, transcription is a massively parallel, intracellular process in which molecular machines, RNA polymerases, unfold short portions of DNA and guide the generation of messenger RNAs. Messenger RNAs, in turn, transfer information to the cell’s protein-making machinery or make other types of RNA that function in a range of enzymatic and regulatory processes. In this manner, RNA polymerases, in conjunction with several other molecular agents, initiate gene expression. Hence transcription errors have effects that cascade throughout the entire G-P mapping process, altering the course of development independently from DNA mutation. Intriguingly, the error rates of transcription and mutation vary dramatically with species, population size, genome size, tissue type, and developmental age; the implications of these variations remains an open and fundamental issue in evolutionary biology (Lynch, 2010). With this in mind, we designed an ECE system that allowed us to control the rate of transcription error, τ, and genetic mutation, μ. In the evolutionary experiments τ and μ are of the same range and magnitude, which facilitates meaningful comparisons between the two different types of noise-making processes.

It’s important to note that transcription error is not the only mechanism in a G-P map that might create an MG effect. As explained below (see Eq. 1), members of the general class of epigenetic developmental errors operate in a similar fashion, by definition, nondeterministically. Nondeterminism, however, could also be present in other elements, including sensors, fitness metrics, and other results of embodied interaction with the agents’ environments that affect the mapping of genes to morphology, performance, and fitness (Arnold, 1983). For example, in evolutionary robotics, fitness evaluations vary across a spectrum, from those that include the investigator’s *a priori* knowledge about the desired result to those that only reward agents for how well they completed a task, independently of how they achieved a given level of performance (Nelson et al., 2009). For any type of fitness evaluation, the presence of nondeterminism at that stage of the mapping may create an MG effect; but randomness in fitness is a very different mechanism than randomness in development, and thus, if experiments support this supposition, it would be tallied as a new type of non-epigenetic, nondeterministic factor (in Eq. 1). Nondeterminism in performance arises from the probabilistic nature of sensors, controllers, actuators, their physical circumstances, and their interactions (Thrun et al., 2005). Moving beyond fitness and performance, nondeterminism in the selection algorithm itself, independent of well-known random evolutionary mechanisms like genetic drift, might create an MG effect. Consider the extreme case in which all individuals, independent of differences in fitness, have an equal chance of reproducing: random mating would inject nondeterminism into this part of the evolutionary process. In the exploratory study reported here, we limit our testing of the MGH to genetic mutation and transcription error, but our model and methodology can also be extended to investigate other nondeterministic mechanisms.

### The ECE Model

In constructing the full ECE model, we were mindful of Vicsek and Zafeiris’ (Vicsek and Zafeiris, 2012) dictum: “A really good model must both reproduce truly life-like behavior and be as simple as possible.” Simple models offer general insights into fundamental processes but lack the details to address specific cases found in nature. Complex models capture natural variations but may create process interactions that are difficult to identify and interpret, creating hidden threats to the model’s validity. Thus we sought to balance the two, choosing simple yet biologically realistic features and processes to model life-like genetics, development, behavior, and evolution of embodied, locomoting individuals. We provide an overview of the ECE model here; details may be found in the Methods section.

Starting with genetics, the single-stranded genome of each agent is 16 kilobases long, with a variable number of genes, from 6 to 55, depending on the evolutionary history of the individual. Both genetic features are similar to those found in RNA viruses such as influenza (Eisfeld et al., 2015). Further, the genetic code uses the codon, a triplet of quaternary digits defined in the biological case by the classic A, T, G, C nucleotide bases, along with redundancy of the code. Start and stop codons demarcate open reading frames, called genes in this study, and their variable positions permit the number and length of coding and non-coding regions to vary between genomes and over generational time. When expressed, each codon produces one or more transcripts, a process that initiates development. Some codons affect the expression of other genes, creating opportunities for epistatic gene interactions.

Development is present, explicit, and simple, with transcripts making five types of protoparts, determining the duration of the growth of the protoparts into finished parts, and defining some properties of the parts. By a set of fixed rules, those parts are assembled into the embodied robot. The body is composed of two or more spheres, creating a simple segmented robot; segments are connected by hinges that may be powered to bend in response to a stimulus from touch sensors that is propagated through a simple neural network of at least one complete sensorimotor circuit. The locomotor behavior is simple terrestrial locomotion on a flat surface without barriers. Each population is composed of 60 individuals. In a given generation, differences in locomotor performance are used to select the top half of the population for reproduction *via* a fixed truncation process. Reproduction is simple and asexual, with mutation possible at any of the base pairs.

This ECE model was built to conduct experiments on the G-P map in embodied, locomoting individuals that are part of evolving populations. Specifically, we systematically covaried the rates of genetic mutation and transcription error. The rate of point mutations, μ, during reproduction were varied from 0 to 5 × 10^–4^ per gene per generation, the maximum rate falling in the range of that measured in RNA viruses, 10^–3^ to 10^–6^ (Lynch, 2010). The rates of transcription error, τ, during gene expression were varied over the same range as μ, facilitating direct comparisons of the quality and magnitude of the two different types of noise. Moreover, a fully factorial experimental design allowed for examination of the interactions of those two different processes in the G-P map.

### Masquerading Genomes From Stochastic Ontogeny (SO)

By altering the G-P map, SO creates what one can think of as masquerading genomes (MG). In our model, the developmental mechanism of SO is transcription error, which creates MG by hiding the deterministic fitness of the genome. Sharing motivation with Stanton (Stanton, 2018), though with a different experimental design, we formally represent every individual *i* as having an evolutionary fitness, *ω*_*i*_, partitioned into two components: deterministic and non-deterministic fitness. If the environment around the developing individual is fully deterministic, as is the case in our experimental paradigm, then the combination of the genome and the epigenetic error, where transcription error is but one type of epigenetic developmental error, can be viewed as fully determining the individual’s evolutionary fitness:

$$\omega_{i}=f\left({\left[genome\right]}_{i},{\left[epigenetic\;errors\right]}_{i}\right)$$ (1)

where [*genome*]_*i*_ is the genome of individual *i* and [*epigenetic errors*]_*i*_ represents the total epigenetic error affecting the development of individual *i*. If all epigenetic errors are null, then it is the case that both the surrounding environment and the individual’s development are fully deterministic, which results in the special case of deterministic fitness. When some epigenetic errors affecting the individual are non-null, *ω*_*i*_ results from a nondeterministic process. Note that any processes resulting in the formation of the genome—including random mutational errors—occur prior to the individual’s development and determination of fitness, and can thus be considered to be part of deterministic fitness. In the nondeterministic fitness case studied in our experiments, when random epigenetic processes (e.g., transcription errors) are operating, these nondeterministic processes can change the *ω*_*i*_ and create a MG.

With evolutionary experiments that systematically covary τ and μ, our model shows that by adding a nondeterministic SO component to the deterministic process of the G-P mapping, transcription errors create MGs. MGs, in turn, increase both genetic and phenotypic variance of the population. The evolutionary experiments were conducted with an 11 × 11 factorial design, with the independent variables τ (11 levels) and μ (11 levels), producing 121 different conditions. Each condition has ten replicates: ten different populations of 60 robots evolved over 100 generations. In every population and every generation, each individual robot is tested in a dry terrestrial world, a simple flat plane in which *ω*_*i*_ is measured as the Euclidean distance that the robot locomotes from start to stop. Selection was directional and constant; populations that undergo adaptive evolution have higher mean fitness, a result of their collective improvement in locomotion.

These experiments produced 7.26 × 10^6^ individuals. For each individual, we tracked 11 properties: identification, parentage, generation, fitness, genetic variance (two types), and number of body parts: joints, neurons, sensors, and wires. This yielded an enormous database of nearly 8 × 10^7^ points. With a data set of this size, the opportunities for analysis are manifold. In this initial work, we develop the ECE model and present a high-level examination of MGs, specifically the influence of μ and τ on genetic variance, *H*, of the population. Unlike the additive genetic variance that must be inferred in biological populations, we can measure genetic variance directly using the Hamming distance between every pair of individual genomes in a given population. The sum of all pairwise Hamming distances, *H*, serves as our measure of the genetic variance of the population.

## Methods

This ECE modeling system consists of three bioinspired models that work in concert: 1) a genetic system that encodes morphological and regulatory traits as triplet codons, mutates the genomes, and replicates the genome for reproduction; 2) a developmental system that expresses over time the genome as sets of transcripts, creates random errors in the transcripts, processes those transcripts to create finished parts, and then uses a fixed set of rules to assemble the parts into individual robots; and 3) an evolutionary system that tests the behavioral performance of the individuals in a population with a physics simulator and selects the best for reproduction.

### Genetic System

**FIGURE 1:**
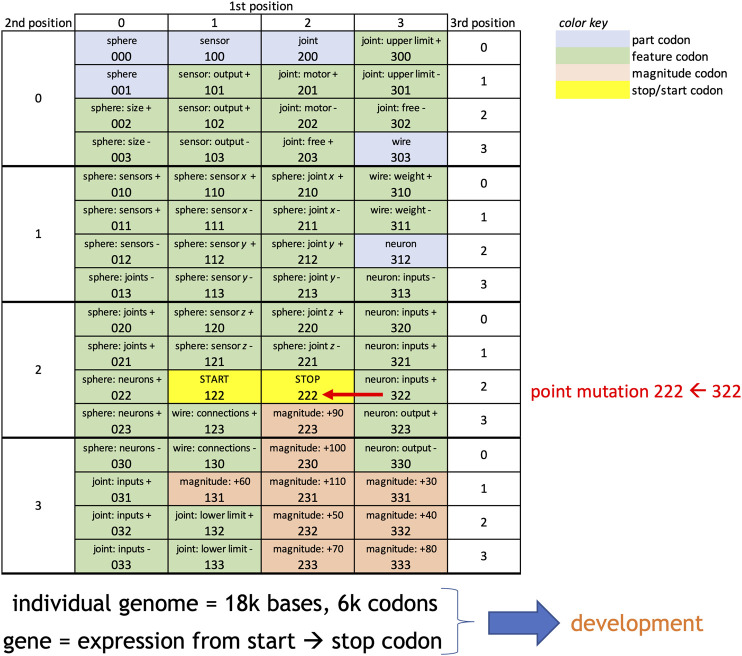
Genetic code and mutation errors for embodied robots. Inspired by the genetic code of lifeforms, this genetic code uses triplet codons to signify parts, features of the parts, magnitudes of the parts, and start and stop codons. In an individual, these codons are expressed as transcripts (see Figure 2) if they occur between the start and stop of a gene. Each individual has a genome that is 18k bases long, allowing for 6k possible codons and multiple genes. Point mutations are random. They occur by changing, for example (see red text), the first digit of the codon from 3 to 2, which changes the codon from 322 to 222, turning a feature codon into a property codon. Point mutations occur only when the genome of a parent is replicated to make the genome of an asexually reproduced offspring.

The robotic genome was designed to mimic certain properties of biological genomes. The biological genome uses the codon, a triplet of quaternary digits defined in the biological case by the classic A, T, G, C nucleotide bases. Each codon is expressed as an RNA transcript; each transcript maps to a particular building block (amino acid) from which an organism is constructed during development. Because there are only 21 amino acids but 64 possible quaternary triplets, redundancy in coding occurs when mapping from codons to components. We used a similar structure, substituting digits from 0 to 3 for the nucleotides (Figure 1).

In generation 0, each genome was initialized as a random string of 6,000 codons (18,000 quaternary digits). Codons fall into four categories (Figure 1): part, feature, magnitude and start/stop. There are five different part types: spheres, joints, sensors, neurons, and wires. Feature codons are the most numerous. In combination they determine, for example, the size of the finished sphere part (feature codons 002 and 003). Magnitude codons are not part specific; they combine to control the relative duration of developmental processes. The *start* and *stop* codons are not expressed; they delimit the beginning and end of a gene. Only codons inside of the *start-stop* boundaries are expressed. The genome is mutated only during reproduction, which follows selection and occurs before the development of the next generation of robots (see *Evolutionary Experiments* section). For each digit in the genome, the mutation operator uses a random number generator and, based on the mutation rate for that population, alters the digit.

In each gene there may be one or more part codons. Only the first part codon after the start codon was expressed. In contrast, all feature and magnitude codons in the gene were expressed, and they formed a pool of regulatory elements. The interaction of the pool of expressed regulatory elements with the expressed part type determined the final size and configuration of the part.

### Developmental System

**FIGURE 2:**
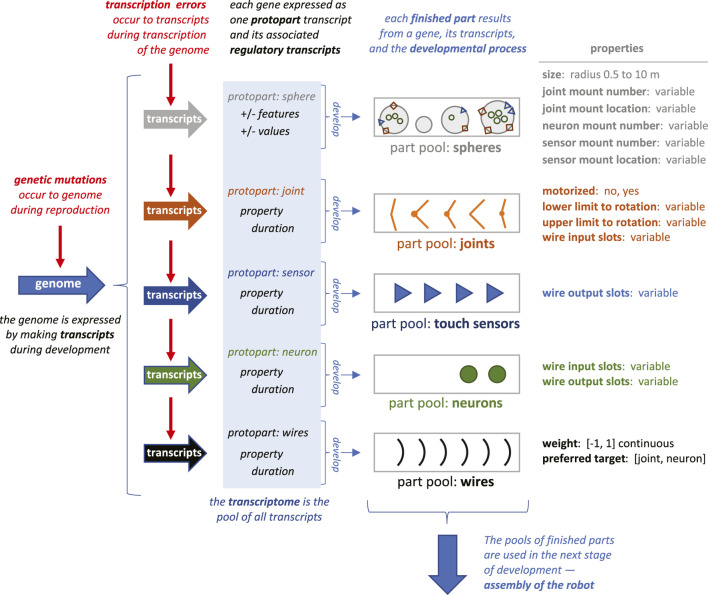
Transcription errors and their impact on early development. Each gene is expressed once during development, making one transcript that is a protopart and multiple transcripts that encode the part’s properties and developmental duration. The protopart develops into a finished part by having property transcripts add to or subtract from the growing part’s properties incrementally over time. The duration of the process—and thus the extent of the incremental effects—is determined by the magnitude regulatory elements (see Figure 1). As parts are finished, they are added in sequence to the individual’s part pool. Early development is complete when all the expressed protoparts have been turned into finished parts. Shapes of parts as represented here are simplifications of the 3D shapes simulated. Not all properties of the parts are represented graphically.

In development, the genotype-to-phenotype (G-P) mapping began with the expression of each gene as a set of transcripts based on the triplet codons. These transcripts specified the protoparts and regulatory elements to be used in building the robot (Figure 2). At this initial stage of gene expression, transcription errors alter the digits of the codon with a probability determined by a random number generator and the transcription error rate for that population. An essential feature of these transcription errors is that they are epigenetic, occurring to products of the genome, leaving the genome itself unaltered.

As in biological systems, the set of transcripts, the transcriptome, is used by the developmental process to build the agent over time, where time is represented by update cycles. The G-P mapping unfolds in time by taking each gene’s protopart and growing it into the finished part, with the duration of the growth and functional properties of the part determined by the transcribed regulatory elements (Figure 2). Every transcript has a genetic basis and may contain an epigenetic alteration of that genetic expression. The two types of errors compared in this study impact the same final process: the development of the body parts from protoparts.

The development of body parts involves the expression of codons and the interactions of their transcripts. For example, feature codons 002 and 003 create two counter-acting transcripts, *sphere: size+* and *sphere: size-* (Figure 1). The final size of a sphere depends additively on the total number of transcripts produced. Other pairs of counter-acting codons perform analogous roles to determine the properties of the five types of parts (Figure 2). The expression of these feature codons is regulated by the transcripts of the magnitude codons. For example, the transcript of magnitude codon 230 allows one hundred transcripts from a gene’s feature codons to be expressed. Note that the magnitude codons have only positive values; as transcripts they operate additively to determine the total number of feature transcripts. Each feature codon in the gene is expressed each update cycle until the limit has been reached for the number of feature transcripts set by the pool of magnitude codons. Recognizing the importance of epistasis in cryptic genetic variation (Zheng et al., 2019) we built a type of epistatic gene interaction into our G-P system: every update cycle, each gene randomly contributes 10% of its newly expressed feature and magnitude transcripts to a general pool; each gene then randomly pulls 5% of the transcripts from the pool. This epistasis effect was held constant across trials, since it was not the focus of this initial investigation.

**FIGURE 3:**
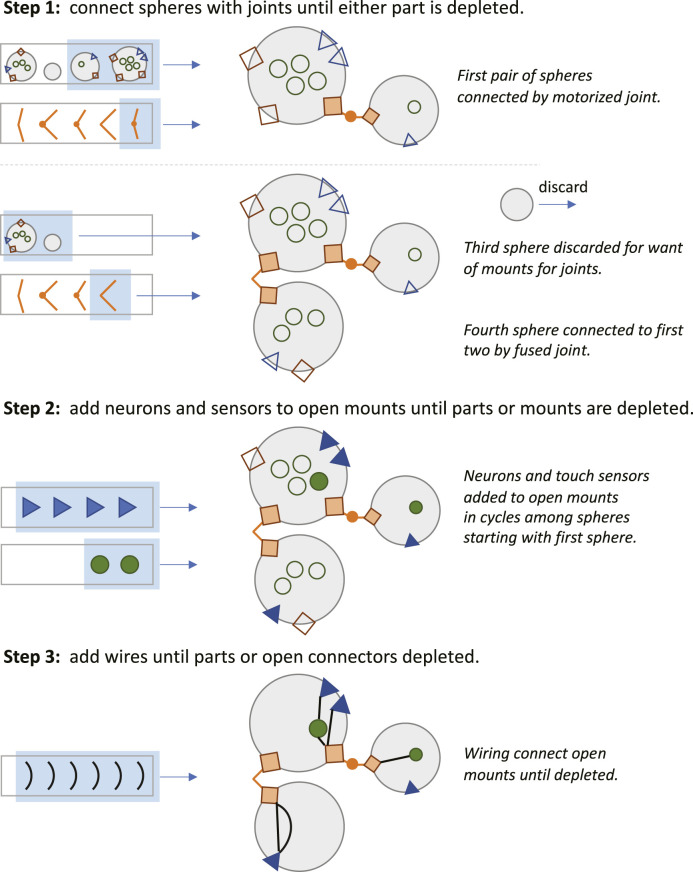
Robot assembly during late development. **Step 1:** Drawing from the part pools (rectangular boxes), development assembles the robot’s body by first connecting a pair of spheres with a joint. This process is repeated until unused spheres, joints, or open joint mounts (empty orange squares) are depleted. **Step 2:** Development adds neurons (filled green circles) and sensors (filled blue triangles) to the mounts on the spheres (empty circles and triangles) until the unused parts or open mounts are depleted. **Step 3:** Unless depleted or without open connections, wires placed to link sensors to neurons, sensors to motors, and sensors to motors.

Time also figures into the assembly stage of development (Figure 3). When a part is finished it is placed in a part pool in the order in which it was constructed relative to other parts of that type (Figure 2). The first part of that type completed was the first from that pool used for assembly of the robot. The rules for assembly specify a building process that takes place in three steps (Figure 3): 1) connect spheres with joints until either part is depleted or when all joint mounts are used, 2) add neurons and sensors to open mounts until parts or mounts are depleted, and 3) add wires until parts or open connectors are depleted. Importantly, development may abort under two conditions: 1) if two or more spheres cannot be created or connected; and 2) if the neural network created by the wires fails to connect at least one sensor to one joint. In either of these cases, the robot cannot move, and it was given a fitness score of 0.

### Evolutionary Experiments

The experiments were organized in a repeated-measures fully factorial design of 11 μ levels and 11 τ levels, for a total of 121 conditions. The error rates for μ and τ were identical values: 0, 0.0005, 0.0010, 0.0015, 0.0020, 0.0025, 0.0030, 0.0035, 0.0040, 0.0045, and 0.0050.

Each population had a fixed size of 60 robots. In generation 0, each genome was generated randomly. There were 10 replicate populations, which means that 600 starting genomes were produced. Each of the 121 conditions started with the same 10 replicate populations. Note that this repeated-measures design, with replicate population as the repeated-measures variable, avoids a confound that would be introduced if all 1,210 populations were different in generation 0.

Each population was evolved for 100 generations with individuals selected for locomotor performance. The simulated environment was flat, dry, empty, and terrestrial. Each robot was given 501 time steps to locomote, and the linear Euclidean distance between its final and initial locations was used as the measure of fitness. This linear distance ignores total length of the path, which, if convoluted, can be substantially greater than the linear distance. Locomotion was driven proximally by the combined action of the robot’s motorized joints in response to stimuli from the robot’s touch sensors. The position of any joint connecting two spheres was the output of the neural network produced in response to input from the touch sensors on the outside of body spheres. The position of each was calculated each time-step of the simulation and normalized to the range [-π, π]. The relative movement of all of the spheres and their interaction with the substrate determined the robot’s overall locomotor behavior (see Supplementry Video S1).

Each population was subjected to truncation selection: the 30 robots with the highest fitness scores reproduced asexually. The three highest-ranked robots each made four children, robots ranked 4–9 each made three children, robots ranked 10–18 each made two children, and robots ranked 19–30 each made one child, resulting in a new population of 60 individuals. In contrast to randomized or random-weighted reproduction algorithms, this fully deterministic method was chosen in part so that the elements of randomness in our design were restricted to only the genetic mutation and epigenetic transcription processes. Truncation selection has two desirable features that are put to use in this study (Crow and Kimura, 1979): 1) it is the most efficient form of directional selection and 2) it reduces the mutational load on the population. The reduction of mutational load likely makes it easier to detect the genetic effects of transcription error.

Bullet Physics libraries (https://bulletphysics.org; v2.82) were used to simulate the robots and the environment. Simulations were run on a system76 Gazelle Professional computer with an Intel^®^ Core™ i7-4710MQ CPUs @ 2.50 GHz. The work of the model was split between two applications: a Python (v2.7.6) program handled development, selection, and reproduction; and a C^++^ program (compiled with g^++^ v4.8.4 using gnu^++^11) simulated robots in the physics engine environment. The random number generators used were RandomState instances from Numpy (v1.8.2).

## Results

**FIGURE 4:**
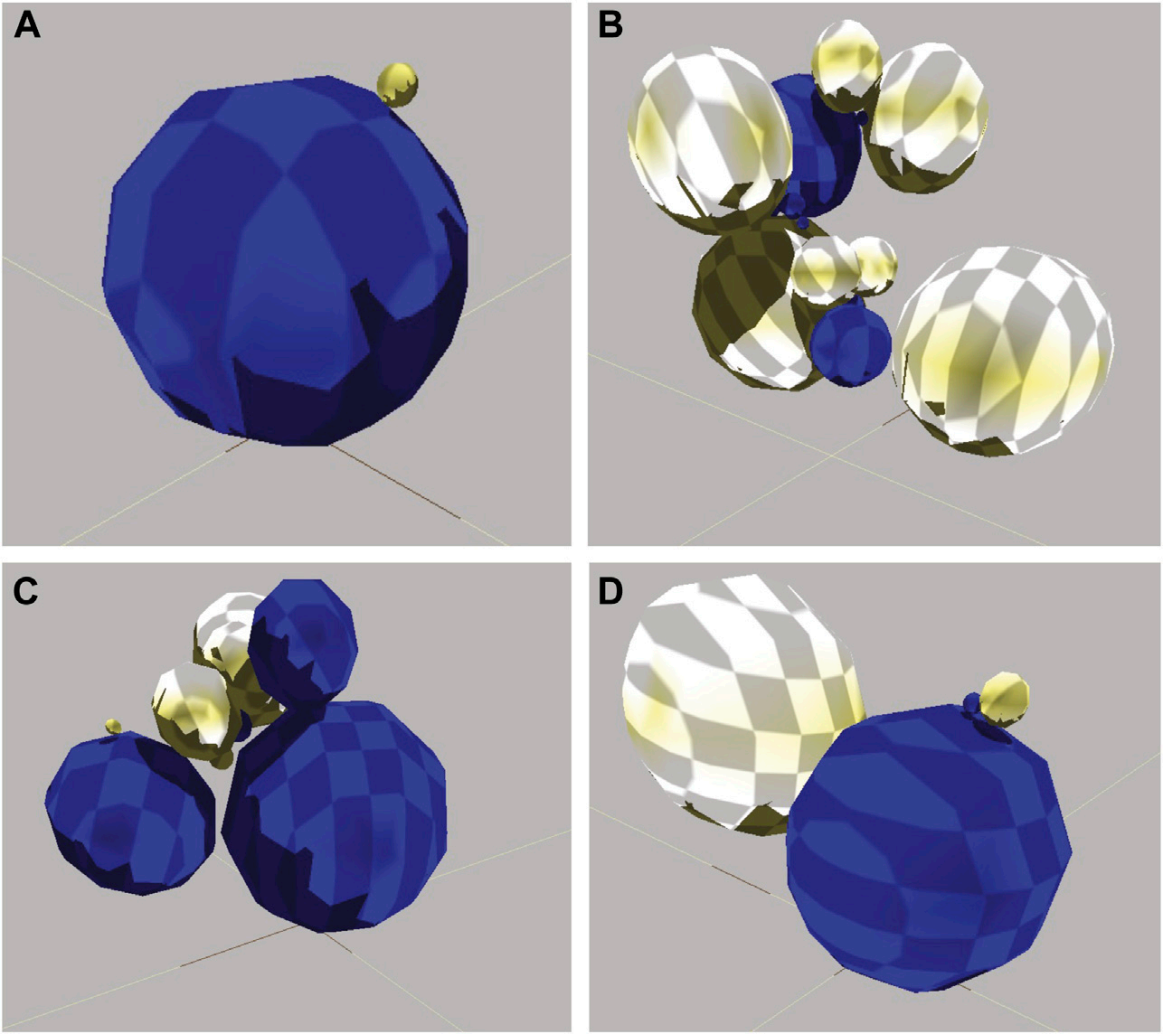
Diversity of evolved robots. **(A)** Simple morphology with low fitness. This is an 8th-generation agent with two spheres, one joint, four neurons, one sensor, and six wires. Lacking a motor, the robot did not locomote. Its non-zero fitness of 0.10 m is an artifact of its large diameter. **(B)** Complicated morphology with low fitness. This is a 92nd-generation robot with 16 spheres, 15 joints, four neurons, five sensors, and three wires. The robot locomoted, but had too many moving parts to do so in an efficient linear fashion. Fitness of 24.49 m. **(C)** Intermediate morphology and fitness. This is a 56th-generation robot with seven spheres, six joints, seven neurons, 10 sensors, and six wires. The robot executed a single jump. Fitness of 199.80 m. **(D)** Simple morphology and high fitness. This is a 83rd-generation robot with four body spheres, three joints, seven neurons, five sensors, and eight wires. It locomoted by continually flipping itself over (see Figure 5). Fitness of 1,068.83 m. Note that of all the parts, only spheres are shown here since their configuration dominates the external morphology.

**FIGURE 5:**
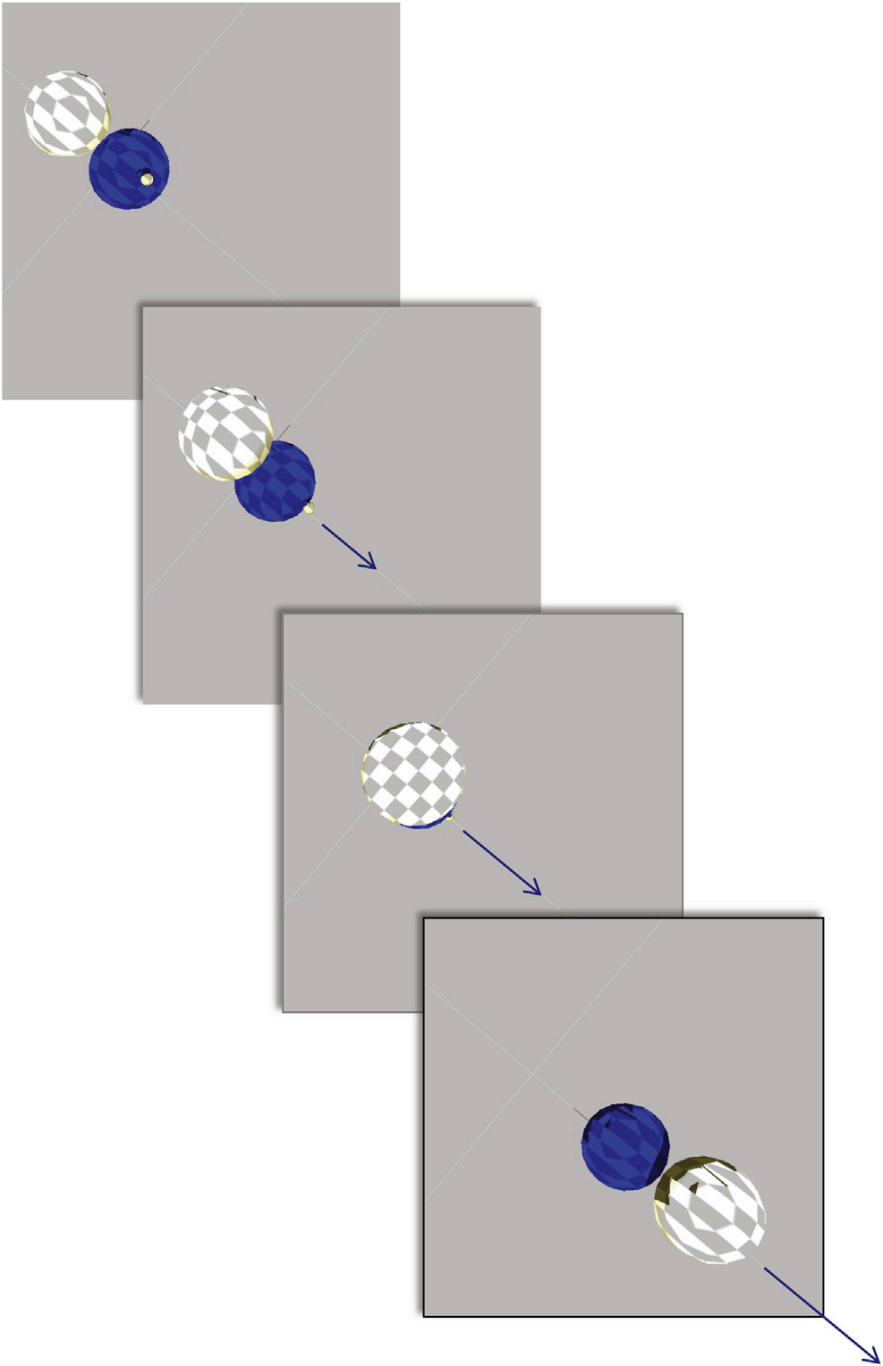
An embodied robot locomoting. View from overhead. Robot from Figure 4D, with high fitness, locomotes by continually flipping one sphere over the other. The arrow indicates both the magnitude and the orientation of the locomotor velocity. Video of robots locomoting is available in supplemental materials.

A total of 1,210 evolutionary runs (10 replicate populations × 121 conditions) of 100 generations produced 7.26 × 10^6^ individual robots. The evolved diversity in morphology ranged from simple two-sphere individuals to those with 16 spheres (Figure 4). The most effective locomotion, and hence highest fitness, was produced by relatively simple morphologies with sensorimotor connections that permitted the rhythmic alternative flipping of the two main spheres (Figure 5). Comparison of the locomotion of three of these individuals is available on Supplementary Video S1.

To test the hypothesis that the rate and pattern of adaptive evolution could be altered by the two independent variables, mutation rate μ and epigenetic error rate τ*,* the mean fitnesses $\bar{\omega }$ of the 1,210 populations were analyzed in two steps. All statistical tests were conducted using SPSS (version 25 on Mac OS 12.12.6). First, the evolution of each population in each condition was summarized by the coefficients from a third-order polynomial regression of individual fitness (60 individuals) onto generation (100 generations): $\bar{\omega }=A+Bx+Cx^{2}+Dx^{3}$. Second, an 11 × 11 (11 levels of μ and τ) repeated-measures ANOVA was run on each of the four regression coefficients. Because the set of ten replicates was identical at the start of all 121 conditions, population was the repeated measures variable. Note that this repeated-measures design avoids a confound that would be introduced if all 1,210 populations were different at the start of evolution.

**FIGURE 6:**
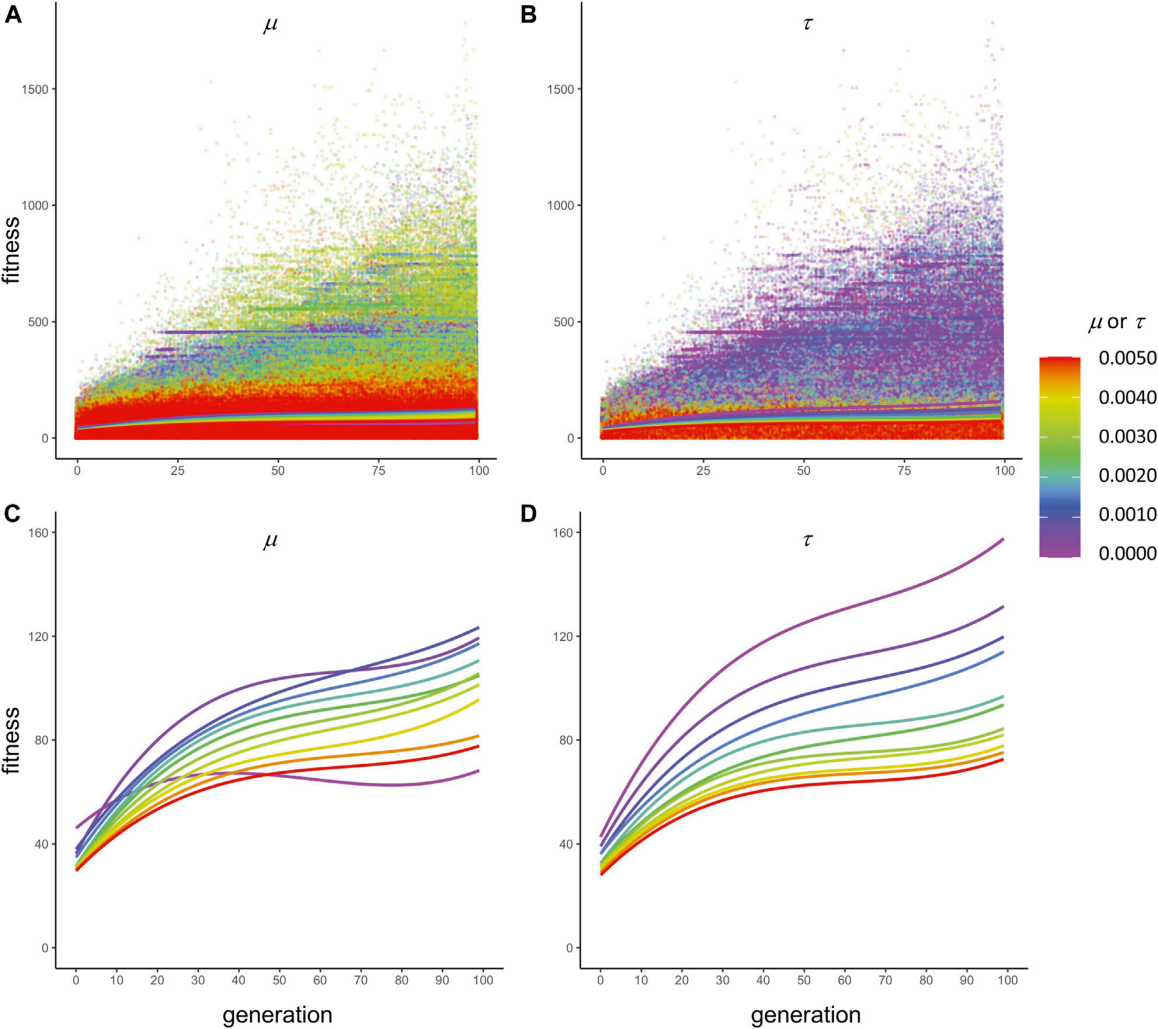
Mutation and transcription error impact adaptive evolution differently. Whether color-coded by the **(A)** rate of mutation, μ*,* or the **(B)** rate of transcription error, τ, the maximum fitness of all 7.26 × 10^6^ individuals in the 10 replicate populations increases over generational time. Note that the only difference between A and B is the color-coding. The polynomial regression lines (3^rd^ order) toward the bottom of A and B are expanded in **(C)** and **(D)**, showing the adaptive trends, with one exception (μ = 0.0000), over time. Mutation and transcription error alter adaptation in very different ways. Low but non-zero levels of μ are associated with the fastest rates of adaptation **(C)**. Populations with μ = 0.0000 (violet curve) show relatively slow and even declining rates of adaptation. Statistically, all mutation effects are significantly linear and not curvilinear (see Table 1). Contrast that with transcription error, where lowest levels of τ (violet) are associated with the fastest rates of adaptation. All transcription error effects are significantly curvilinear (see Table 1). Even though μ lacks statistically significant second- and third-order coefficients, the full third-order model is graphed to facilitate comparison between mutation and transcription error.

The analysis did not detect a statistically significant interaction term for μ and τ on the B, C, and D coefficients of (Table 1); the interaction term for A, the constant in the regression model, was significant. The main effect of μ on $\bar{\omega }$ is significant only for the A and B coefficients, suggesting that the evolutionary effect of genetic mutation on fitness is linear over generational time. The main effect of τ on $\bar{\omega }$ is significant for all four coefficients, suggesting that the evolutionary impact of epigenetics on fitness is more complicated than that of mutation. These results are evidence that changes in μ and τ 1) alter the rate and pattern of adaptive evolution, 2) exert their influences on $\bar{\omega }$ independently, and 3) act on $\bar{\omega }$ in different ways (Figure 6). Most importantly, the non-linear effects of τ on $\bar{\omega }$ show a pattern of initial constant acceleration until generation 20, deceleration until generation 70, followed by secondary acceleration (Figure 6D). The secondary acceleration of $\bar{\omega }$ demonstrates that any convergence that may have begun, indicated by deceleration, was only temporary. This secondary acceleration does not occur over time with the isolated effects of μ (Figure 6C).

**TABLE 1:** Adaptation over 100 generations of directional selection, statistical analysis of regression coefficients in four repeated-measures ANOVAs.

Coefficient	Effect	*F* (df)	*P*	Partial *η* ^*2*^
A	μ	26.92 (4.7, 42.4)	**0.000**	0.749
	τ	26.33 (4.1, 37.2)	**0.000**	0.745
	μ × τ	3.42 (6.0, 53.6)	**0.006**	0.275
	Population	1,170.64 (1, 9)	**0.000**	0.992
B	μ	10.84 (3.5, 31.1)	**0.000**	0.546
	τ	28.45 (4.0, 36.3)	**0.000**	0.760
	μ × τ	1.94 (5.5, 49.6)	0.098	0.178
	Population	421.18 (1, 9)	**0.000**	0.979
C	μ	3.16 (4.2, 37.8)	0.023	0.260
	τ	7.82 (3.7, 33.4)	**0.000**	0.465
	μ × τ	1.41 (5.3, 47.8)	0.234	0.136
	Population	197.42 (1, 9)	**0.000**	0.956
D	μ	1.69 (4.1, 37.3)	0.170	0.158
	τ	4.99 (3.3, 29.7)	**0.005**	0.356
	μ × τ	1.28 (6.1, 56.7)	0.280	0.125
	Population	173.5 (1, 9)	**0.000**	0.951

All tests and degrees of freedom (df) were corrected for lack of sphericity using Greenhouse-Geisser. Population is the repeated-measures variable. To account for four ANOVAs conducted on the same data set, the significance threshold after correction by Bonferroni was 0.0125 (0.05/4). Significant *p* values under Bonferroni are bolded.

**FIGURE 7:**
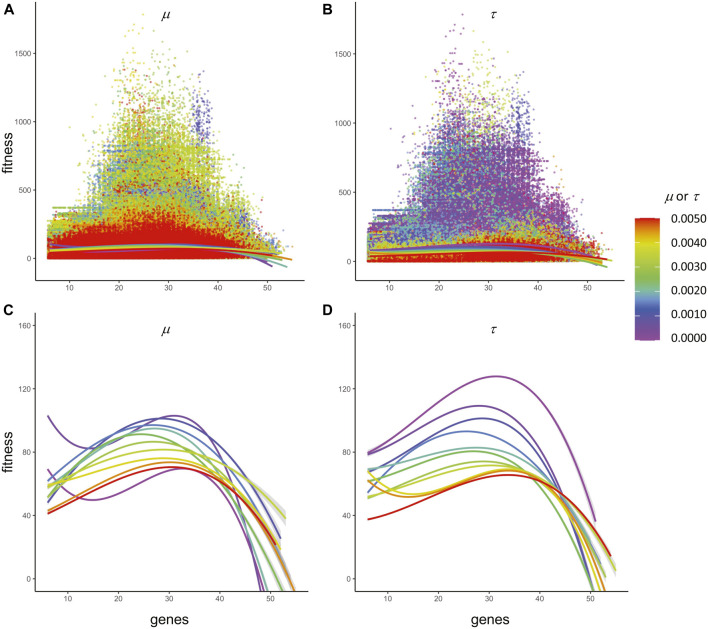
Epistasis creates non-additive fitness. All 7.26 × 10^6^ individuals evolved vary in the number of genes from 6 to 55. Whether color-coded by the **(A)** rate of mutation, μ*,* or the **(B)** rate of transcription error, τ, the fitness of individuals appears to be greatest at intermediate levels of genes. Note that the only difference between A and B is the color-coding. The polynomial fits (3^rd^ order) toward the bottom of A and B are expanded in **(C)** and **(D)**, showing the non-linear relation between number of genes and individual fitness and the different fitness peaks under different rates of μ or τ. The sharp drop-off in fitness as the number of genes increases after the peak demonstrates negative epistasis.

To test for the presence of epistasis—the non-additive interactions of genes—third-order polynomial regression was performed on fitness and the number of genes, grouped by μ and then τ (Figure 7). No matter the grouping or the magnitude of μ or τ, the pattern is similar: strong negative epistasis occurs after a peak of 30–40 genes. At a lower number of genes, simple additive effects are present, and these are not normally considered to be epistasis (Phillips, 2008).

**FIGURE 8:**
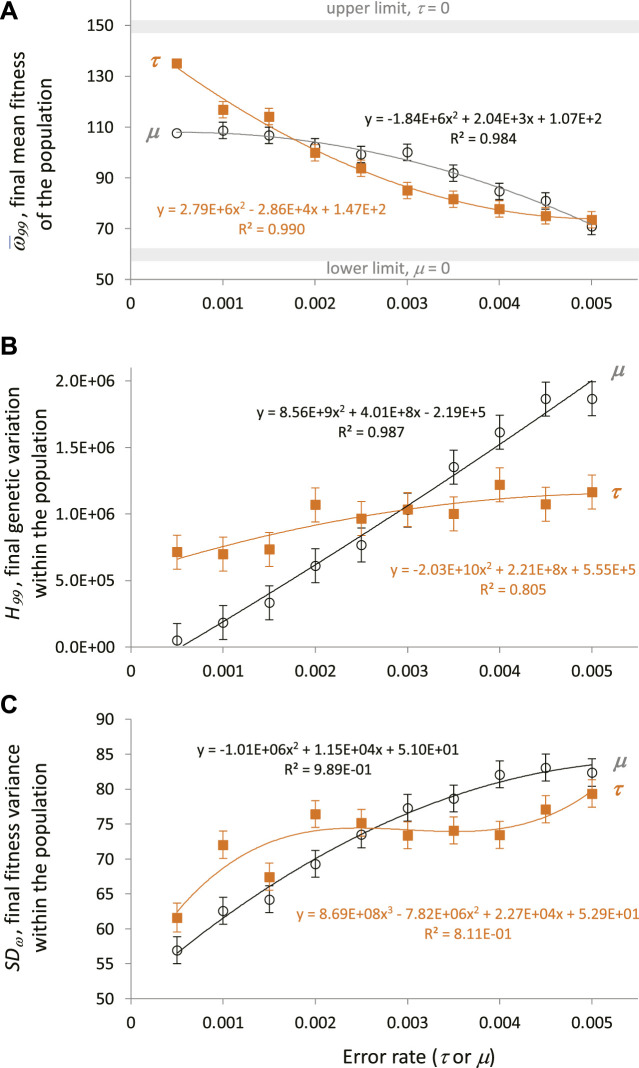
Transcription error and mutation increase genetic variation in the face of directional selection. Analysis of generation 99, the final generation, shows the cumulative impact of continuous directional selection. Points are estimated marginal means ± SE of ten populations generated by the fully factorial 10 × 10 ANOVAs. Polynomial regressions are shown for descriptive purposes only. **(A)** Final mean fitness of the population decreases significantly (main effects, *p* < 0.05) with increasing levels of mutation and transcription error. The interaction of the main effects was not significant. **(B)** Final genetic variation within populations increases significantly (main effects, *p* < 0.05) with increasing levels of genetic mutation and transcription error. The interaction of the main effects was not significant. **(C)** Final fitness variance within a population, as measured by the SD of fitness. Final fitness variation increases significantly (10 × 10 ANCOVA, mean fitness as a covariate) with significantly increasing levels of genetic mutation and transcription error. The covariate and the interaction of the main effects were significant as well (*p* < 0.05).

To investigate the evolutionary outcomes in more detail, the final generation, 99, was examined. For statistical analysis, the conditions with 0 μ and τ were removed because they were outliers by more than 3 SE relative to the nearest values from other conditions; their removal does not alter the qualitative results that follow. To further assess the effects of μ and τ on ${\bar{\omega }}_{99}$, a 10 × 10 ANOVA was performed. Both μ and τ were significant main effects (*p* < 0.05), with the effects size test η_*partial*_ for τ nearly twice that of μ (0.295 v. 0.136). The interaction effect was not significant. To illustrate the nature of the independent effects, the estimated marginal means were regressed against the error rates; as error rates of μ and τ increase, ${\bar{\omega }}_{99}\;$ decreases (Figure 8A). Note that low rates of τ are associated with greater levels of ${\bar{\omega }}_{99}$ than μ at the same levels. The trend is reversed at intermediate error levels, and both converge at the highest error levels.

To test the hypothesis that τ increases genetic variance, *H*, a 10 × 10 fully factorial ANOVA was performed on the final genetic variance, *H*_*99*_. Both μ and τ were significant main effects (*p* < 0.05), with the effects size test η_*partial*_ for μ an order of magnitude greater than for τ (0.223 v. 0.021); the interaction effect was not significant. To illustrate the nature of the independent effects, the estimated marginal means were regressed against the error levels. Results show that as error levels of μ and τ increase, *H*_*99*_ increases (Figure 8B). Note that low levels of τ are associated with greater magnitudes of *H*_*99*_ than μ at the same levels; the range of increased *H*_*99*_ associated with τ is correlated with the relative increase in ${\bar{\omega }}_{99}$ (compare Figures 8A,B).

To test the hypothesis that τ increases phenotype variance, *SD*_*ω*_, measured as the standard deviation of the collection of individual *ω*_*i*_ values for each population, a 10 × 10 fully factorial ANCOVA was performed; ${\bar{\omega }}_{99}\;$ was treated as a covariate to remove the effects of the correlation of *SD*_*ω*_, and ${\bar{\omega }}_{99}$. Both μ and τ were significant main effects (*p* < 0.05), with the effects size test η_*partial*_ for μ three times that for τ (0.176 v. 0.054). The interaction effect was significant as well with an η_*partial*_ of 0.133. To show the independent effects, the estimated marginal means were regressed against the error levels; as error levels of μ and τ increase, *SD*_*ω*_ increases (Figure 8C). Note that low levels of τ are associated with greater magnitudes of *SD*_*ω*_ than μ at the same levels; the range of increased *SD*_*ω*_ associated with τ is correlated with the relative increase in ${\bar{\omega }}_{99}\;$ and *H*_*99*_ (compare Figures 8A–C).

**FIGURE 9:**
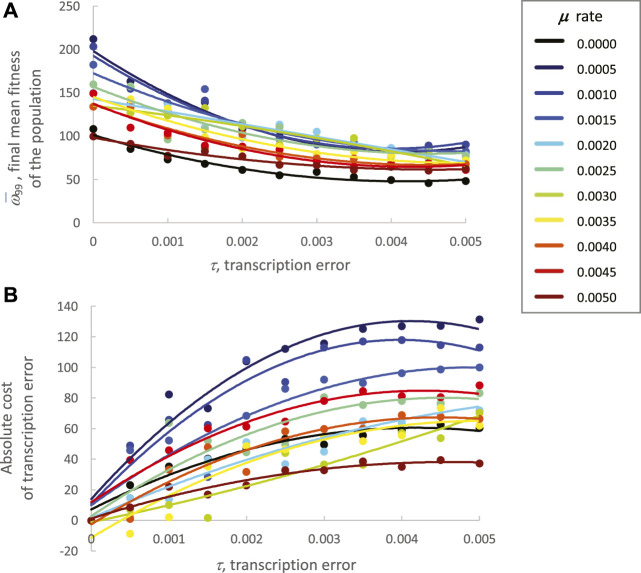
Final mean fitness (generation 99) of the population varies with mutation and transcription error. Note that these data are descriptive, and no statistical tests were conducted. Means are the mean fitness of the ten populations in each condition. Curves are second-order polynomials fit by least-squares regression. **(A)** Final mean fitness. Except for 0.0005 and 0.0010 levels of mutation, as transcription error increases, the final mean fitness of the population decreases. The relationship between rate of mutation and fitness is more complex; low to intermediate levels of mutation, from 0.0005 to 0.0035 cluster consistently with the highest fitness over the range of epigenetic errors. Each point is the mean of all individuals across all ten populations. **(B)** Cost of transcription error. The absolute cost of transcription error, measured as a loss of fitness (difference from fitness at 0 transcription error), is consistently high across levels of transcription error for the three lowest rates of mutation; the smallest loss across levels of transcription error occur in the populations with the highest rate of mutation.

Finally, the fitness cost of μ and τ on ${\bar{\omega }}_{99}\;$ were evaluated (Figure 9). Except for 0.0005 and 0.0010 levels of μ, as τ increases the ${\bar{\omega }}_{99}\;$ decreases (Figure 9A). The relationship between μ and ${\bar{\omega }}_{99}\;$ is more complex; low to intermediate levels of μ, from 0.0005 to 0.0035, cluster consistently with the highest fitness over the range of τ. The absolute cost of τ, measured as a loss of $\bar{\omega }$ (difference relative to $\bar{\omega }$ at τ_0_), is consistently high across the range of τ for the three lowest μ; the smallest costs across levels of τ are the populations with the highest μ (Figure 9B).

Data for $\bar{\omega }\;$ and *H* are available upon request. Please note that data on the evolution of body parts are being evaluated for a separate publication and will be released when that work is in print.

## Discussion

Our simulated embodied computational evolution (ECE) system develops individuals and evolves populations of embodied and behaviorally autonomous robots using bioinspired features and mechanisms. Each individual possesses a genome of 18,000 quaternary bases encoding information in triplet codons (Figure 1). Development begins with the expression of an individual’s genes into transcripts that map information about body parts and the assembly of those parts onto a process of construction (Figures 2, 3). In each generation, the morphologically variable adults (Figure 4) are tested in a locomotor task, with the relative individual fitness scores determining differential reproduction in the population. Selected individuals reproduce asexually with random genetic mutations of the genome transmitted to the offspring. As offspring develop, epigenetic errors occur as errors of transcription. This ECE system allows researchers 1) to model and to experiment on the mapping of the genotype to the phenotype (G-P map) of simulated, embodied populations that interact with their environments, and 2) to address questions about the relative influence and interaction of genetic and developmental processes on the evolution of populations of mobile agents. These are abiding, fundamental issues in evolutionary systems for which the ECE approach offers traction.

In building this system, we were guided by a foundational question in evolutionary disciplines from evolutionary biology to evolutionary robotics: How has biological evolution created an enormous variety of lifeforms? Workers in evolutionary robotics have shown that the action and interaction of selection and mutation are insufficient 1) to avoid premature convergence on adaptive peaks and 2) to expand the diversity of behaviors and morphologies [for review, see (Stanton, 2018)]. Evolutionary biologists have arrived at the same conclusion by comparing genomic with quantitative genetic approaches (Charlesworth, 2015). In response to the limitations of selection and mutation, workers have developed algorithms that search for novelty and diversity (Lehman and Stanley, 2008; Lehman and Stanley, 2011; Brawer et al., 2017) and introduce stochastic ontogenetic noise into the G-P map (Stanton, 2018). To help understand these alternative evolutionary mechanisms and their interactions with selection and mutation, this current work tested the following hypothesis: Random epigenetic error increases genetic variation in evolving populations.

To test this hypothesis we evolved populations of embodied robots under directional selection, covarying levels of genetic noise created by random mutation, μ, and levels of epigenetic noise created by random transcriptional error, τ. The μ and τ were of the same range and magnitude, which facilitated meaningful comparisons between the two different types of random error-making processes. We selected for improved locomotor behavior while tracking mean fitness, phenotypic variation, and genetic variation in the populations. We ran 121 different pairwise combinations of μ and τ in 10 different starting populations of 60 individuals over 100 generations. The results were clear: random errors in transcription increase genetic variation (Figure 8B).

As verified by the experimental results (Table 1; Figure 6), transcriptional errors act independently from genetic mutation, providing an additional degree of freedom for researchers engaged in evolutionary search. The functional difference between these two mechanisms can be characterized by their effects on the relation between a genome and its fitness (Eq. 1): epigenetic errors introduce a nondeterministic process in the mapping of genotype to phenotype and the phenome to fitness. The nondeterminism creates masquerading genomes (MG) that “hide” genetic variation behind a phenotypic “mask.” In this way, epigenetic errors create cryptic genetic variation, which can be a critical feature of populations and their ability to respond adaptively to changes in selection (Zheng et al., 2019).

While it is tempting to think of this cryptic genetic variation as solely neutral in the classical sense of a point mutation in a redundant position in a codon, the effect of cryptic genetic change—deleterious, neutral, or beneficial—depends on how that genotype is mapped through development. In the case presented here, if the nondeterministic effects of transcriptional errors are present, then the phenotypic effect of the genetic change—whatever its valence—is randomized with respect to the individual’s fitness (Eq. 1). With higher rates of transcriptional error, the accumulation of mutations is not prevented by selection; this makes the system robust to mutations and increases its long-term evolvability (Masel and Trotter, 2010). The trade-off for mutational robustness is an immediate reduction, relative to populations with less robustness, in the population’s mean fitness and response to selection (see next paragraph). In this ECE model, we did not directly test for the benefits of mutational robustness; if mutational robustness were in play, a random change in environment would have greater negative effects on populations with lower rates of transcriptional error. But one could extend this model to let the rate of transcriptional error itself evolve. When rates of mutation are high and mean fitness of the population is depressed, selection may restore fitness by evolving mutational robustness (Franklin et al., 2019); thus in the case of this ECE model, we would predict that the rate of transcriptional error would increase in response to high, fixed mutation rates. This would be a test of the “survival of the flattest” hypothesis, where so-called “flat” genotypes, with low fitness and high robustness, may out-compete “fit” genotypes, which possess high fitness and low robustness (Wilke et al., 2001; Franklin et al., 2019).

As predicted by the mutational robustness hypothesis, MGs in this ECE system and their cryptic genetic variation incur a cost: as the magnitude of τ increases, the mean fitness, $\bar{\omega }$, of the population decreases (Figures 8A, 9). This loss of fitness is offset in two ways: 1) genetic variance is increased (Figure 8B) and 2) phenotypic variance is increased (Figure 8C). Increasing variance of both types enhances the response of populations to selection, which acts on phenotypes directly and genotypes indirectly. It is crucial to note that mutation as an engine of genetic variation also incurs a similar cost: as the rate of mutation, μ, increases, the population’s mean fitness decreases (Figure 6C). Like transcription error, high μ serves to keep both genetic (Figure 8B) and phenotypic (Figure 8C) variance high after 99 generations of selection. While the costs and benefits are similar by the measures of population variance, it is worth keeping in mind that mutation scrambles adapted genomes but transcription errors do not. This difference at the level of individuals should lead to differences in the populations’ long-term adaptation and is the reason that we predict that the MG effect is, in fact, an example of mutational robustness (Wagner, 2005). Indeed, transcription errors cause the evolutionary trajectories to accelerate their adaptation late in our experiments, near generation 90 (Figure 6D); recall that the second-order coefficients that offer the statistical evidence for that late acceleration in $\bar{\omega }$ are significant for τ but not for μ (Table 1). We did not measure whether that acceleration is a continuation of local convergence or the discovery of a different adaptive peak.

Even with these costs, populations under steady selection retain the ability to explore, avoid final convergence, and find new solutions. First, the parallel adaptation of 121 types of population, differentiated by the magnitude of τ and μ, shows a variety of evolutionary trajectories that probe the search space in different ways (Figure 6). Second, the statistically isolated impact of the epigenetic errors on the pattern of serial adaptation for any single population over generational time is curvilinear: an initial increase in τ slows and then accelerates (Figure 6B). These parallel and serial effects work, in part, because τ maintains and increases genetic variation even when μ is low and selection is steady and directional (Figure 7B). Thus random epigenetic errors alter the evolutionary behavior of populations in principled ways that augment the tools we have to investigate adaptation and diversity.

We note that random transcription error is but one possible type of developmental epigenetic error. Stanton (Stanton, 2018) added Gaussian noise to the evolved weights of neural networks, which increased absolute fitness under a variety of environmental conditions in simulated quadrupedal robots. Our robotic system and experimental design differed from that of (Stanton, 2018) in a number of ways; most importantly, the selection pressure and environment were held constant while μ and τ were systematically covaried. Moreover, our system showed a trade-off in fitness with increasing epigenetic error rather than the clear benefit in Stanton (2018), a difference that we are eager to explore. Finally, by covarying both types of error our experimental protocol allows the direct comparison of the evolutionary consequences of genetic and epigenetic error.

The importance of epigenetic errors in adaptive exploration signals the general importance of modeling an explicit relationship between the genotype and the phenotype as mediated by development and its feedback effects on evolution. This relationship unfolds over developmental time as the G-P mapping process. By including genetically encoded regulatory elements (feature and capacity codons, Figure 1), the system presented here allows for the G-P process itself to evolve. This aspect of our system is thus consistent with calls to acknowledge and permit developmental systems, and not simply adult phenotypes, to be the focus of evolutionary investigations (Northcutt, 2002; Pfeifer and Bongard, 2006). The evolutionary importance of development, all else being equal, is demonstrated by the significant impacts of the transcription errors on adaptation (Figure 6) and genetic and phenotypic variation (Figure 8).

### Design Decisions and Scope

When interpreting the results of these experiments, it is important to keep in mind a number of important limitations of this ECE model. Some of the most consequential decisions made in the formulation of the ECE model are highlighted here. We recognize that those decisions foreclose the exploration of other mechanisms and effects; with this in mind, we offer several ideas for further studies, as well as ideas for studies of mechanisms and effects not in the immediate focus of this research.

*Simplifications and abstractions.* This ECE model of genetics, development, behavior, and evolution is a simplification of even the simplest biological systems. While the genome size and number of genes are similar to the magnitude seen in RNA viruses, the expression of those genes in our model initiates a cascade of just a few processes relative to those seen in cells (Figures 2, 3). Moreover, developmental processes are abstracted away from their chemical foundations, as is seen in the post-translational assembly of the body, which occurs deterministically (Figure 3). Furthermore, the simple bodies of the robots, with only touch sensors for inputs, eliminates the possibility of evolving novel goals with respect to the environment. The environment, for that matter, is flat and featureless, lacking objects, other agents, and any detectable stimulus gradients. The benefit of these simplifications and abstractions is that they permit a clear analysis of how variations and interactions of mutation and transcription error impact the evolutionary dynamics of a population of simulated, embodied, locomoting robots.

Beyond these simplifications and abstractions, the tight focus of this study’s experimental design—on mutation, transcription error, and their interactions—leaves other mechanisms unexplored. As noted in the Introduction, the problem for the scientist is that nondeterminism impacts all or nearly all parts of the mapping of genotype to fitness and the population’s collective evolutionary response. Thus we recommend that decisions about what nondeterministic factors to explore depend on the hypothesis to be tested. For example, an evolutionary roboticist might hypothesize that in terms of producing MG effects, aggregate fitness evaluations are equivalent to behavioral fitness evaluations (Nelson et al., 2009) when the latter includes nondeterminism. The experimental design might focus, as we have done here with transcription error, on covarying the putative causal mechanism—the amount of *a priori* deterministic information in the fitness evaluation—with a primary genetic operator, mutation, leading to a two-way factorial statistical design. In another example, a biologist might borrow from the field of genetic algorithms (Whitley, 1989) to hypothesize that since selection is, by definition, a deterministic mechanism, otherwise identical populations of embodied individuals can generate different evolutionary responses based solely on their differences in reproduction.

*Population size.* The fluctuation and magnitude of population size play key roles in evolutionary dynamics. Each of the ten replicate populations was coerced in our reproduction algorithm to be of constant size (n = 60). In organisms, population size often fluctuates wildly, with small numbers creating bottlenecks that dramatically reduce genetic variance and effective population size, key parameters in extinctions (Gillespie, 1998). While holding size of the population constant may be unrealistic, a small population size is not; it is often the case that mere tens of individuals are seen in founder populations or populations nearing extinction. But it is important to recognize that in populations of fewer than 100 haploid individuals, reductions in population size drastically alter the critical mutation rate, the threshold above which individuals with greater mutational robustness are favored over individuals with greater fitness (Aston et al., 2013). In our system of 60 haploid, asexual individuals, we did not measure critical mutation rate and so cannot address whether our intentional changes in mutation rate shifted the population from the evolution of the fittest to the evolution of the “flattest” (Wilke et al., 2001; Franklin et al., 2019). Both fluctuating population size and changes in critical mutation rate offer intriguing opportunities for the next set of experiments using this ECE model.

*Selection*. When interpreting the results of these evolutionary experiments, it is important to keep in mind that we applied the most efficient form of directional selection: truncation (Crow and Kimura, 1979). We chose a fixed threshold of 50% fitness of the population, and thus the genomes of 50% of the population are not represented. When τ is zero, truncation selection would remove genomes that map deterministically to phenotype and to fitness, creating the largest possible evolutionary response *ceteris paribus*. This is what we see (Figure 6): as τ increases, the evolutionary response to selection decreases, as seen in the reduced slopes of the polynomial regressions at any particular generation. This reduced response is caused by the blurring of the truncation threshold, as some less fit genomes are selected because their phenotypes have been relatively improved by epigenetic transcriptional error. Thus the rate of transcriptional error is a principled way to alter truncation selection from a step function to a more gradual threshold. It is worth noting that other forms and patterns of selection exist that are likely to alter the evolutionary dynamics that we see here. For example, disruptive and stabilizing selection would, respectively, increase and reduce the genetic variation of the population by focusing selection on different parts of the fitness distribution. Moreover, at the level of choosing individuals, probabilistic methods, like roulette wheel reproduction, would further act to blur the clear line of truncation selection.

*Time, equilibrium, and convergence*. For each of the 1,210 runs (10 replicate populations run in 121 experimental conditions), a total of 100 generations were run. It is important to recognize that 100 generations is an arbitrary window. At the same time, natural populations of locomoting vertebrates show rapid and significant adaptive changes in a few to tens of generations (Stuart et al., 2014; Reznick et al., 2019).

Related to the issue of the number of generations (see previous paragraph) is the lack of equilibrium or convergence of any of the populations over generational time (Figure 6). While it is important in evolutionary computation to have algorithms that find local optima in the search space, we had a different goal: Our primary focus was on computational methods for simulating the evolutionary behavior of living systems, to gain insight into incompletely understood aspects of biological evolution. It is worth noting that natural populations are open dynamical systems, inherently unstable (Gregorius, 2001). Thus by demonstrating only temporary stability near generation 50, our populations show the kind of on-going change that we expect out of rich natural systems. As with any real population, it would be intriguing to extend this model further into its own evolutionary future.

*Epistasis and the evolution of genes*. Epistasis, the interaction between genes, is a ubiquitous phenomenon in living systems, but one that makes it daunting to understand the role of individual genes (Phillips, 2008). Genes in natural systems operate as part of an interacting, fluctuating genetic network, and we have captured some of that complexity in our ECE model. Epistatic effects were part of every agent’s developmental system—10% of every gene’s feature and magnitude transcripts were placed in a pool from which other genes drew (see *Developmental System* section, Methods). These coded epistatic effects were held constant in our experiments manipulating τ and μ. We therefore had anticipated that they would be a constant factor in the evolutionary system. Indeed, as measured by the relation of the number of genes to fitness, epistatic effects are similar across the experimental treatments (Figure 7). But we did not anticipate the strong negative epistatic effects that are seen when the number of genes is greater than 30 or 40. Clearly, something interesting is occurring.

The analytical challenge in understanding epistasis is that the number of interactions scales with the square of the number of genes (Phillips, 2008). Hence, for every individual agent, which may have as many as 55 genes, there may be over 3,000 interactions as it develops within our system. In addition, keep in mind that the ECE model does not fix the number or size of genes; both are free to evolve as indicated by the genetic code (Figure 1), a variation that complicates analysis. In future studies, the ECE system could be modified to track the interactions among genes by source-tagging the feature and magnitude codons that move into the collective pool. Moreover, one could manipulate the amount of epistasis as an independent variable to test the open hypothesis that epistasis slows evolutionary change (Phillips, 2008). The more daunting issue is conceptual: how does one understand an interaction between a pair of genes that is part of a larger interaction network that includes not just the genomic map but also the G-P map and its mapping onto fitness? ECE modeling offers a principled way to investigate those molecular dynamics while retaining the essential larger context of embodied individuals behaving autonomously as part of a population that is evolving.

## Summary

The novel system described here creates populations of simulated, embodied robots that operate with 1) a genetic system that encodes morphological and regulatory traits; 2) a developmental system that expresses over time the mapping between genotype and phenotype, allowing for epigenetic errors in the process; and 3) an evolutionary system that tests the behavioral performance of the individuals in a population and selects the best for reproduction. The explicit dynamics of this system take place on all three temporal scales of evolution (Franklin et al., 2019): behavior, development, and evolution.

Evolutionary and developmental mechanisms balance and adjust the adaptive and exploratory behavior of populations. Mutation, the sole genetic operator modeled here, and transcription error, the sole epigenetic operator, are independent and complementary mechanisms—they adjust the rates of adaptation, as measured by the change in mean fitness of the population, with lower levels of both errors associated with higher initial rates of adaptation (Table 1; Figure 6). When considering only the final mean fitness of the populations, their impact is dramatic (Figure 8). Small, non-zero transcription errors are associated with higher fitness than mutation of the same magnitude (Figure 8A); as transcription error and mutation increase, fitness falls, with populations possessing intermediate levels of mutation having greater fitness than those with comparable levels of transcription error.

Armed with epigenetic transcription errors, the evolving populations not only counteract the tendency of directional selection to remove genetic variance, but they can also increase that genetic variance. Systematic exploration of those epigenetic errors demonstrates a new mechanism to be used in conjunction with selection and mutation for the exploration of the adaptive landscape.

## Data Availability

The raw data supporting the conclusions of this article will be made available by the authors, without undue reservation.

## Footnotes

1 There are different usages of the term *embodied*; we use it in the sense in the sense popularized by Pfeifer et al. (2005), in which embodied agents are those “realized as physical systems.” This meaning may include simulated robots, such as those used here, provided that their bodies and their environments interact according to the dynamics of physical laws.

2 The term “epigenetic” varies widely in meaning. We use it here in Waddington’s original sense, the processes between the genotype and phenotype (Dupont et al., 2009; Deichmann, 2016), with emphasis on those processes having no direct effect on the DNA sequence of the individual in which they occur.
